# First detection of *Enterocytozoon bieneusi* in whooper swans (*Cygnus cygnus*) in China

**DOI:** 10.1186/s13071-020-3884-y

**Published:** 2020-01-07

**Authors:** Yuexin Wang, Kaihui Zhang, Yifan Zhang, Ke Wang, Azhar Gazizova, Luyang Wang, Letian Cao, Yajun Zhang, Jianying Huang, Yuan Cui, Yuxi Zhang, Longxian Zhang

**Affiliations:** 1 College of Animal Science and Veterinary Medicine, Henan Agricultural University, Zhengzhou, Henan People’s Republic of China; 2 Sanmenxia Management Office of Henan Yellow River Wetland National Nature Reserve, Sanmenxia, Henan People’s Republic of China; 3 Sanmenxia Swan Lake National Urban Wetland Park Management Office, Sanmenxia, Henan People’s Republic of China

**Keywords:** *Enterocytozoon bieneusi*, *Cygnus cygnus*, Zoonotic, Genotype, Migration

## Abstract

**Background:**

*Enterocytozoon bieneusi* is a parasite that infects humans and a wide range of other animals. The large migratory waterfowl, the whooper swan (*Cygnus cygnus*), travels through many cities during its migration and can spread parasites. Despite receiving increasing attention worldwide, there have been no reports of *E. bieneusi* infection occurring in *C. cygnus*. Therefore, this study aims to assess the prevalence and genetic characteristics of *E. bieneusi* in *C. cygnus* in Sanmenxia, China.

**Methods:**

Altogether, 467 fresh fecal samples were collected in the Swan Wetland Park in Sanmenxia, China. Genomic DNA was extracted from fresh fecal samples (*n *= 467) and *E. bieneusi* was identified by nested PCR amplification of the internal transcribed spacer (ITS) region. ITS-positive sequences were aligned and phylogenetically analyzed to determine the genotypes of *E. bieneusi*.

**Results:**

The overall prevalence of *E. bieneusi* in *C. cygnus* was 7.49% (35/467). Sequencing of the 35 positive samples revealed eight known genotypes (EbpA, EbpC, Henan-III, Henan-IV, BEB6, CD9, Peru6 and PtEb IX) and three novel genotypes (CSW1, CSW2 and CSW3). The phylogenetic tree constructed from the ITS sequences showed that seven genotypes (Peru6, EbpA, EbpC, Henan-III, CSW3, Henan-IV and CSW1) clustered within the zoonotic Group 1 while the remaining novel genotype CSW2 clustered within Group 5.

**Conclusions:**

To our knowledge, this is the first report of *E. bieneusi* in *C. cygnus*. Of public health significance, our results suggest that migratory *C. cygnus* might play an important role in the water-borne transmission of *E. bieneusi*. Effective strategies will be necessary to control *E. bieneusi* infection in *C. cygnus*, other animals and humans.
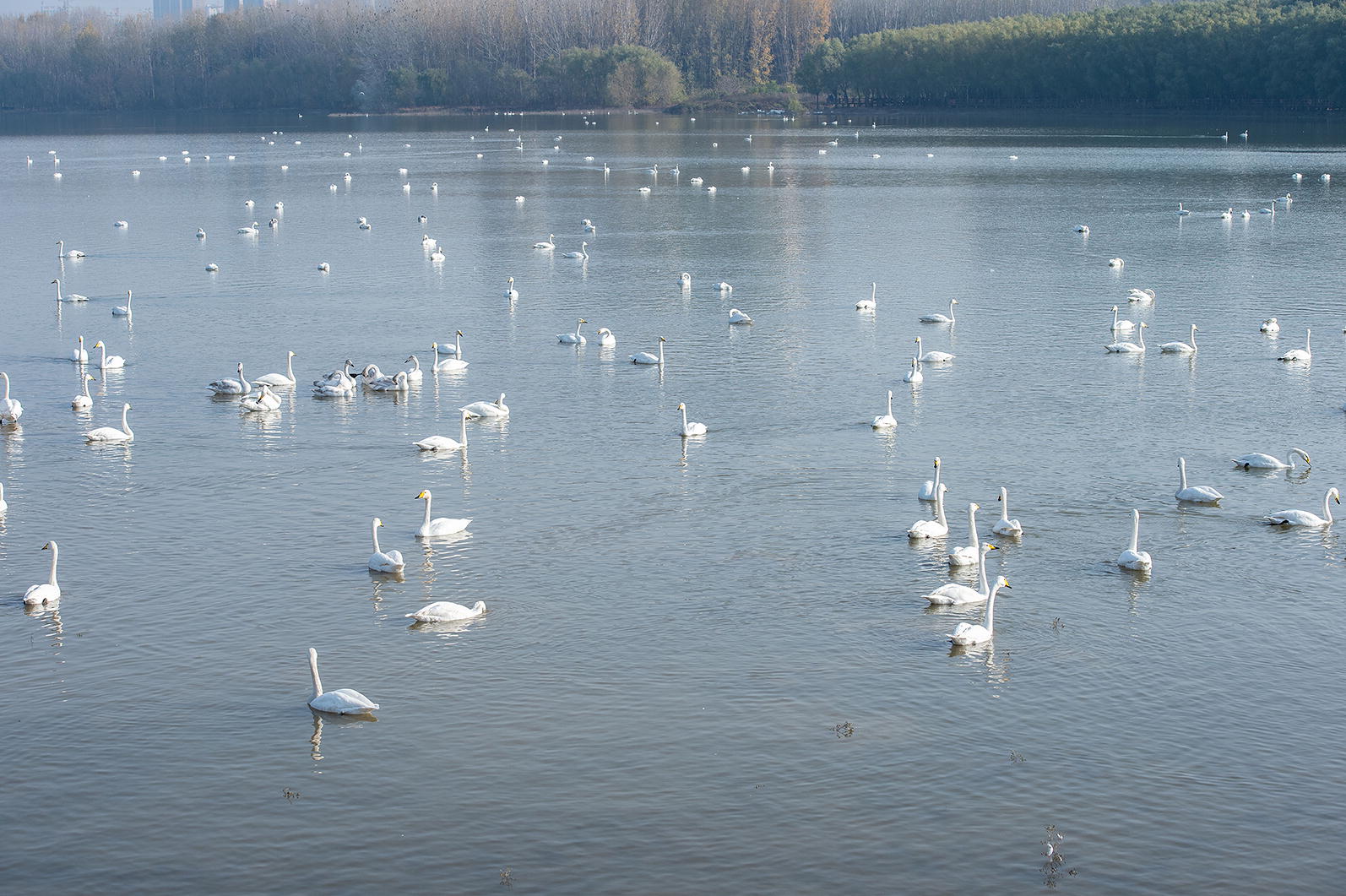

## Background

Microsporidia are thought to be zoonotic parasites with the capability of infecting hosts from all major animal taxa worldwide [1]. Of the approximately 1200 species of microsporidia that have been named [2], 17 can infect humans [3]. Up to now, *Enterocytozoon bieneusi* has the highest worldwide prevalence and is responsible for more than 90% of human microsporidiosis cases [4]. Microsporidia can infect immunodeficient individuals, including those infected with the human immunodeficiency virus and cancer patients. There have been increasing reports of *E. bieneusi* infections in people with immune disorders, or with sub-optimal immunity such as children and the elderly [5]. There are many ways that *E. bieneusi* can be contracted, including from food and water. Furthermore, many genotypes that can infect both humans and domestic animals have been identified making microsporidiosis a zoonotic disease [6].

Currently, PCR-based methods are widely used to detect *E. bieneusi* and to analyze its molecular genetic characteristics [2]. Nested PCR and sequencing of the internal transcribed spacer (ITS) region of the ribosomal rRNA gene cluster have identified 474 *E. bieneusi* genotypes globally [6]. Through phylogenetic analysis, *E. bieneusi* genotypes can be divided into 11 groups. Genotypes in Group 1 (e.g. EbpA, EbpC, Peru6, D and type IV, among others) usually infect humans and other animals, with some risk of zoonotic transmission. Some genotypes in Group 2 (e.g. BEB6, BEB4, I and J, among others) were originally classified as ruminant-specific [7] but are believed to have developed reduced host specificity because these genotypes have been identified in other hosts. Significant public health concerns have been raised in connection with the zoonotic potential of these genotypes.

Recently, many *E. bieneusi* genotypes in Group 1 have been identified worldwide in birds, and these birds may play essential roles in pathogen spread [6]. However, there have been no reports of *E. bieneusi* infection occurring in the whooper swan, *Cygnus cygnus*. *Cygnus cygnus* (order Anseriformes, family Anatidae) are large migratory birds with long migratory paths. These birds travel through cities, forests, reservoirs and lakes during migration. To lay the foundations for future studies, the present study employed three fecal sampling sessions timed before the migration of *C. cygnus* (November and December 2018, and March 2019) to investigate the prevalence of *E. bieneusi* in these birds. We also determined the migration route taken by the winter-migratory *C. cygnus* from Sanmenxia, China. If *C. cygnus* become infected with *E. bieneusi*, they could pose a health threat to other animals including humans, as well as to the environment along their migration pathways. Hence, we carried out this study to assess the prevalence and genetic characteristics of *E. bieneusi* in *C. cygnus* in Sanmenxia, China. Our findings will support protection strategies for *C. cygnus* and public health strategies to address the threat of the zoonotic spread of *E. bieneusi*.

## Methods

### Sample collection

**Fig. 1:**
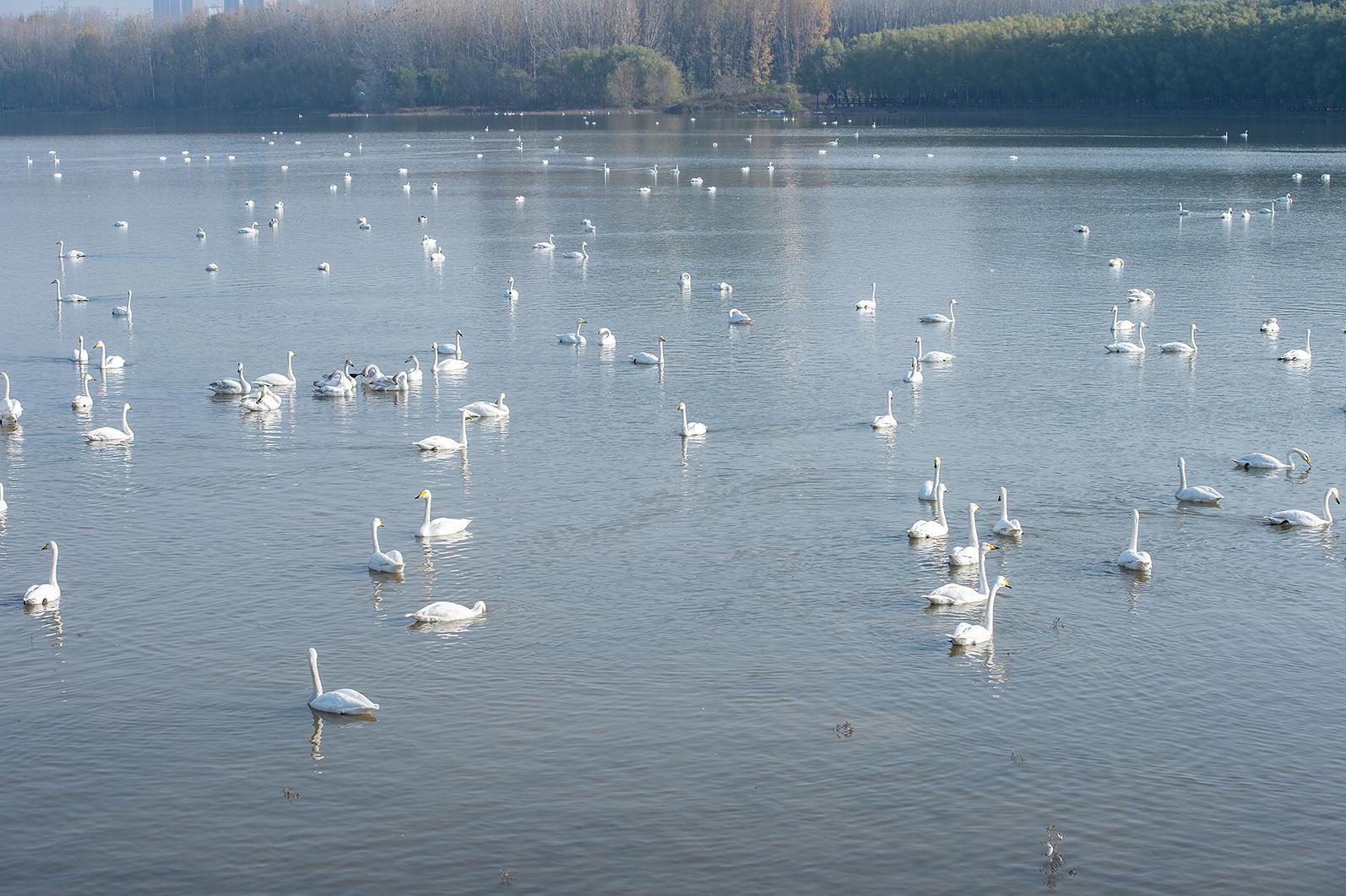
Whooper swans (*Cygnus cygnus)* in the Swan Wetland Park in Sanmenxia, China

**Fig. 2:**
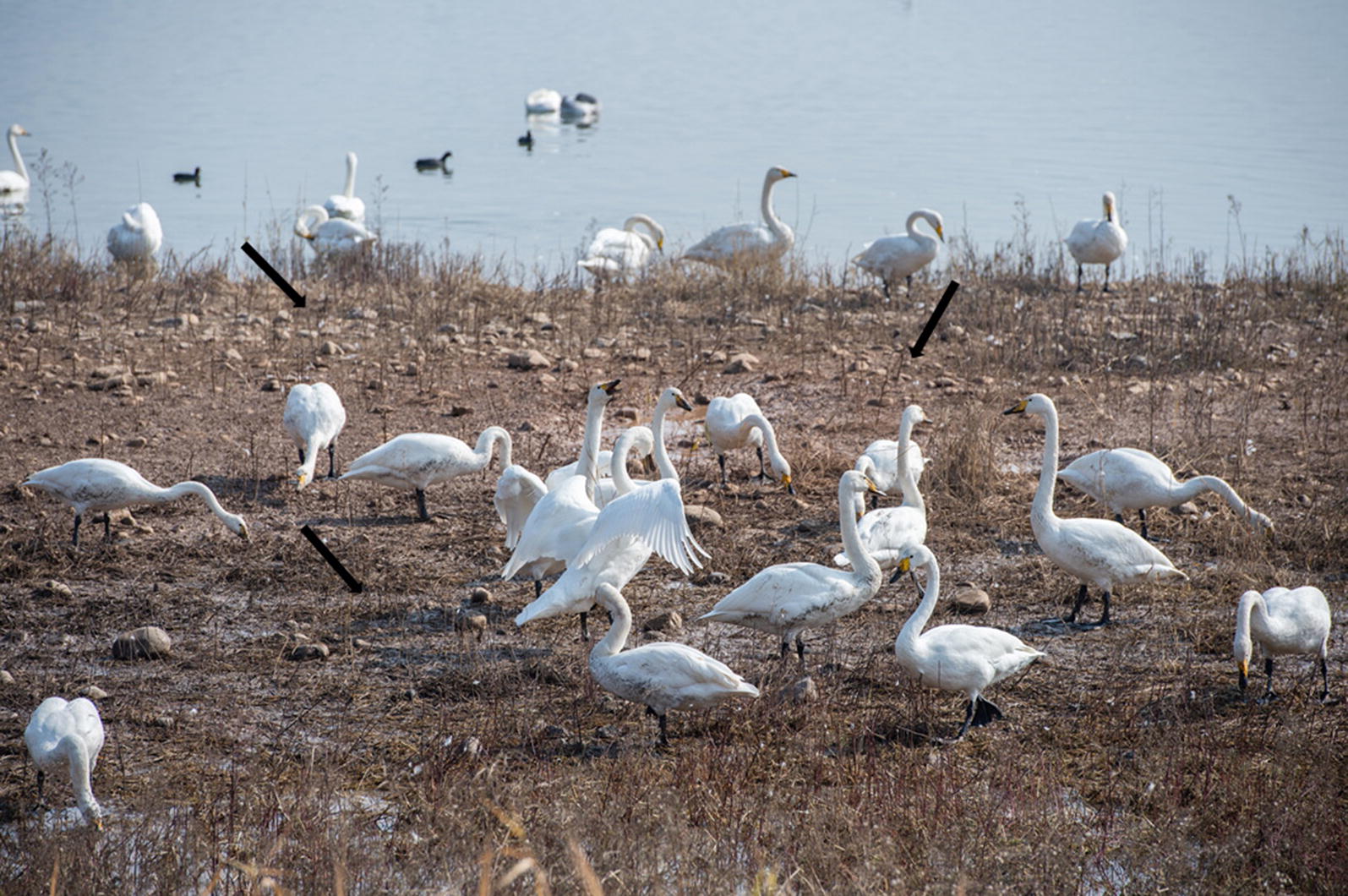
Sample locations (black arrows) in the Swan Wetland Park in Sanmenxia, China

Altogether, 467 fresh fecal samples were collected during three sampling sessions in Sanmenxia, Henan. The first batch of 237 samples was collected in November 2018, the second batch of 161 samples was collected in December 2018 and the third batch of 69 samples was collected in March 2019. The time of these three sampling periods was in the winter when *C. cygnus* started wintering or were about to migrate and their numbers were highest. We collected feces from an island in the lake during the feeding period of *C. cygnus* to ensure the samples were fresh (Figs. 1, 2). We then marked and recorded the color and shape of the fecal samples. Approximately 30–50 g of fresh fecal sample (not touching the ground) was collected immediately into a disposable plastic bag after defecation using a sterile disposal latex glove. All fecal samples were stored in 2.5% potassium dichromate at 4 °C until processing.

### DNA extraction

Each fecal sample was washed at least three times with distilled water and centrifuged at 3500 ×*g* for 5 min to remove potassium dichromate. Before DNA extraction, we transferred up to 200 mg of each stool sample to a 2 ml microcentrifuge tube containing 200 mg of glass beads. The samples were then vortexed at maximum speed until the stool samples were completely homogenized. DNA was extracted using the E.Z.N.A. Stool DNA Kit (Omega Biotek Inc., Norcross, GC, USA) in accordance with the manufacturerʼs instructions. The extracted DNA was aliquoted and stored at− 20 °C.

### PCR amplification and sequence analysis

Nested PCR and sequencing of the ITS region of *E. bieneusi* rRNA gene cluster was used to identify genotypes. We used the primer sequences described by Buckholt et al. [8]. The external PCR primers were EBITS3 (5′-GGT CAT AGG GAT GAA GAG-3′) and EBITS4 (5′-TTC GAG TTC TTT CGC GCT C-3′) and the internal PCR primers were EBITS1 (5′-GCT CTG AAT ATC TAT GGC T-3′) and EBITS2.4 (5′-ATC GCC GAC GGA TCC AAG TG-3′). rTaq DNA polymerase (Takara Bio Inc, Shiga, Japan) was used for PCR amplification. All PCR amplifications included both a positive and negative (no DNA) control and were performed in triplicate. All positive amplification products were confirmed with bidirectional sequencing to ensure accuracy. The sequences were edited using Clustal X, version 2.1 (http://www.clustal.org), Chromas Pro, version 2.1.5.0 (http://technelysium.com.au/ChromasPro.html) and were then aligned with reference sequences downloaded from GenBank. Phylogenetic trees were constructed using the neighbor-joining method (Kimura 2-parameter model) using MEGA, v.7.0 [9], and a bootstrap analysis with 1000 replicates was used to assess tree reliability. The nucleotide sequences generated in this study were deposited in the GenBank database under the accession numbers MN179305-MN179315.

### Statistical analysis

Prevalence differences between different collection times were analyzed using the Chi-square test in SPSS (Release 13.0 standard version; SPSS Inc., Chicago, IL, USA). Values of *P* < 0.05 were considered statistically significant. The 95% confidence intervals (CI) of the prevalence were also calculated by SPSS for Windows.

## Results

### Prevalence of *E. bieneusi* in *C. cygnus*

The overall prevalence of *E. bieneusi* in *C. cygnus* was 7.49% (35/467, 95% CI: 5.10–9.89%, *χ*^2^ = 0.693, *df* = 2, *P *> 0.05) across the three sampling sessions (Table 1). There were no significant differences in prevalence across the three sampling sessions [first: 8.44% (20/137, 95% CI: 4.87–12.00%), second: 6.83% (11/161, 95% CI: 2.89–10.77%), third: 5.80% (4/69, 95% CI: 0.14–11.45%)].

**Table 1:** Prevalence and genotypes of *E. bieneusi* in whooper swans (*Cygnus cygnus*) in Sanmenxia, China

Collection date	*n*/*N*	Prevalence (95% CI) (%)	Genotype
November 2018	20/237	8.44 (4.87–12.00)	EbpC (*n *= 7); EbpA (*n *= 7); Henan-IV (*n *= 1); BEB6 (*n *= 1); PtEb IX (*n *= 2); CSW1 (*n *= 1); CSW2 (*n *= 1)
December 2018	11/161	6.83 (2.89–10.77)	Peru6 (*n *= 3); EbpC (*n *= 2); BEB6 (*n *= 1); CD9 (*n *= 1); Henan-III (*n *= 1); PtEb IX (*n *= 2); CSW3 (*n *= 1)
March 2019	4/69	5.80 (0.14–11.45)	Peru6 (*n *= 3); CD9 (*n *= 1)
Total	35/467	7.49 (5.10–9.89)	EbpC (*n *= 9); EbpA (*n *= 7); Peru6 (*n *= 6); PtEb IX (*n *= 4); Henan-IV (*n *= 1); Henan-III (*n *= 1); BEB6 (*n *= 2); CD9 (*n *= 2); CSW1 (*n *= 1); CSW2 (*n *= 1); CSW3 (*n* = 1)

*Abbreviations*: n, number positive; N, number examined

### Genetic characterization and genotype distribution of *E. bieneusi* in *C. cygnus*

We identified 11 *E. bieneusi* ITS genotypes, including eight known genotypes (BEB6, EbpC, EbpA, Peru6, Henan-IV, Henan-III, PtEb IX and CD9) and three novel genotypes (CSW1, CSW2 and CSW3) (Table 1). EbpC was the predominant genotype (*n* = 9) causing 25.7% of *E. bieneusi* infections, followed by EbpA (20.0%) and Peru6 (17.1%). The genotypes EbpC, PtEb IX and BEB6 were detected in both the first and second sampling sessions, and genotype Peru6 was detected in both the second and third sampling sessions.

### Phylogenetic analysis

**Fig. 3:**
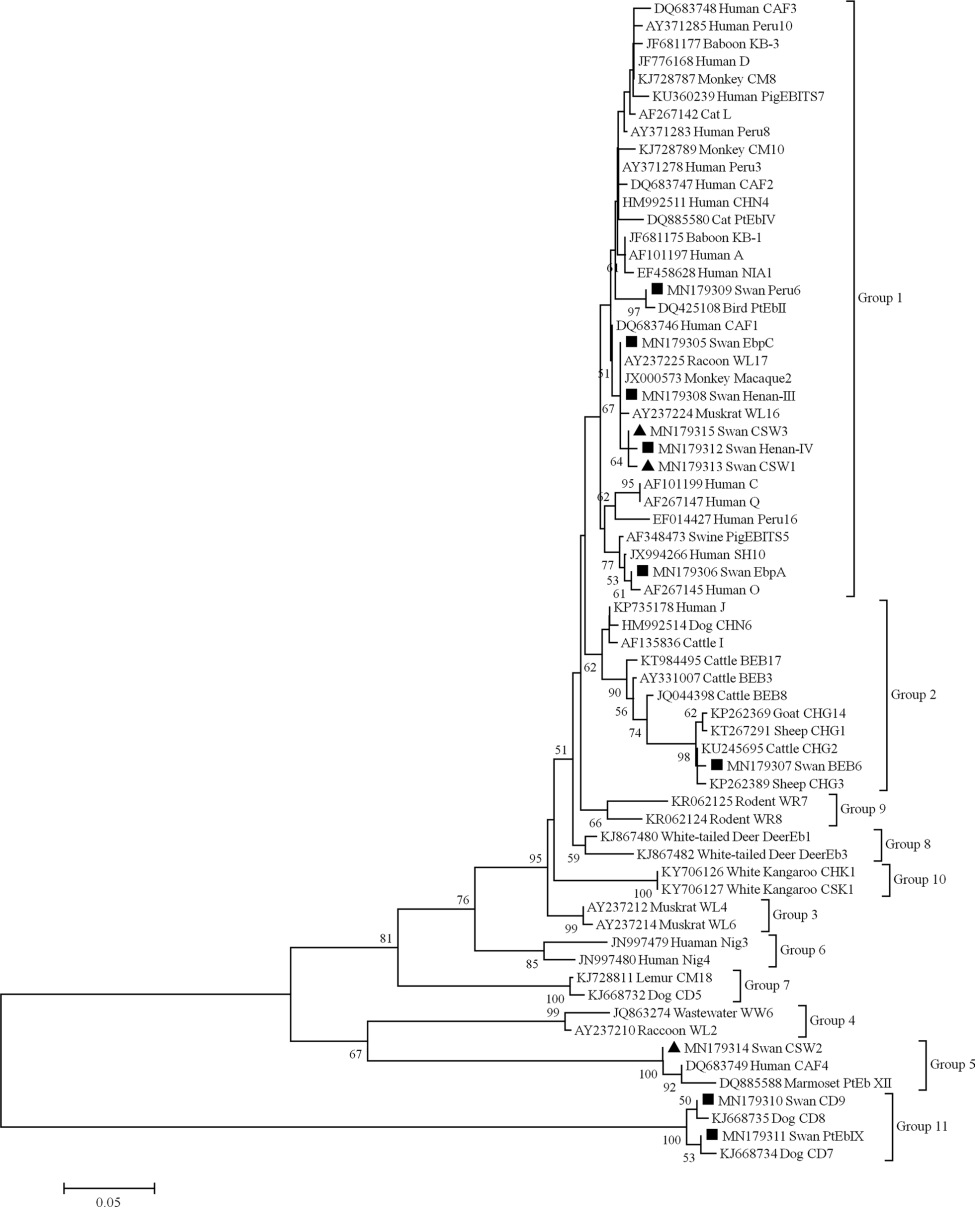
Phylogenetic relationships among *E. bieneusi* isolates inferred from ITS sequences using the neighbor-joining method. Known and novel genotypes are indicated by filled triangles and squares, respectively

According to the phylogenetic tree constructed using the ITS sequences from the known novel genotypes identified in this study as well as reference sequences, the genotypes EbpA, EbpC, Henan-III, Henan-IV, Peru6, CSW1 and CSW3 belonged to Group 1. Genotype BEB6 belonged to Group 2 and genotype CSW2 belonged to Group 5. The remaining genotypes (CD9 and PtEb IX) all clustered within Group 11. In this study, we also identified three novel genotypes (CSW1, CSW2 and CSW3). Compared with Henan-IV (GenBank: JQ029727.1), CSW1 contains two single nucleotide polymorphisms (SNPs) in the ITS region (C23T and G107T). Two SNPs were also identified in the ITS region (G43A and G62A) from genotype CSW3 with genotype Henan-IV (GenBank: KJ728794.1), and CSW2 contains one SNP in the ITS region (A32G) with KB-6 (GenBank: JF681180.1) (Fig. 3).

## Discussion

In this study, we found that the overwintering *C. cygnus* prefers open, food-rich and shallow waters such as lakes rich in aquatic plants, ponds and slow-flowing rivers, especially in the boreal forest. It has been reported that *E. bieneusi* had a wide-host range that includes humans, domestic animals and wild animals [3]. However, *E. bieneusi* has not been reported in *C. cygnus* to date. To the best of our knowledge, our study is the first to show the common occurrence of this pathogen in *C. cygnus*. We found an overall prevalence of 7.49% of *E. bieneusi* in overwintering *C. cygnus* in Sanmenxia, China. Prior to our study, da Cunha et al. [10] had found *E. bieneusi* in a swan goose from a Brazilian market. However, there is only one study of waterfowl, similar to swans, that reported the prevalence of *E. bieneusi* in cranes (12.5%, 7/56), ducks (9.7%, 6/62) and geese (30.8%, 8/26) in China [11]. The prevalence of *E. bieneusi* in *C. cygnus* is likely to be affected by multiple factors, including the health of the host, the environmental conditions, and the specificity of the detection methods used [3]. Therefore, it is difficult to explain the different prevalence values reported in [11]. Hence, more research is needed to investigate the situation of migratory animals and waterfowl infections with *E. bieneusi*.

The nucleotide sequence analysis of the ITS region from the *E. bieneusi* isolates revealed the presence of 11 genotypes including eight known genotypes (EbpA, EbpC, Henan-III, Henan-IV, Peru6, BEB6, CD9 and PtEb IX) and three novel genotypes (CSW1, CSW2 and CSW3). Interestingly, in the present study, EbpC, a zoonotic genotype, which has been detected worldwide in humans and in domestic and wild animals such as pigs, cattle, deer, sheep, dogs, horses, pandas, mice, foxes, beavers and raccoons [6], had the highest prevalence. The genotype EbpA, which has previously been identified in humans in Nigeria and China [12, 13] and is widely reported in porcine worldwide [8, 14–16], was also found to be highly prevalent. Moreover, two genotypes (Henan-III, Henan-IV) from the present study have only been found in humans, pigs and non-human primates in China [17–19], whereas Peru6 and BEB6 have been identified in various birds, ducks and geese [11]. This is also the first time that known zoonotic genotypes have been reported in *C. cygnus*, indicating that this species may play an important role in the transmission of *E. bieneusi*.

Based on the phylogenetic relationships among *E. bieneusi* isolates, as inferred from the ITS sequences, two genotypes (CSW1 and CSW3) fell into Group 1 indicating that they may imply a potential zoonotic risk. However, we found no significant differences in prevalence between the three collection time-points. Therefore, we suspect that *C. cygnus* may act as a reservoir host of *E. bieneusi.*

**Fig. 4:**
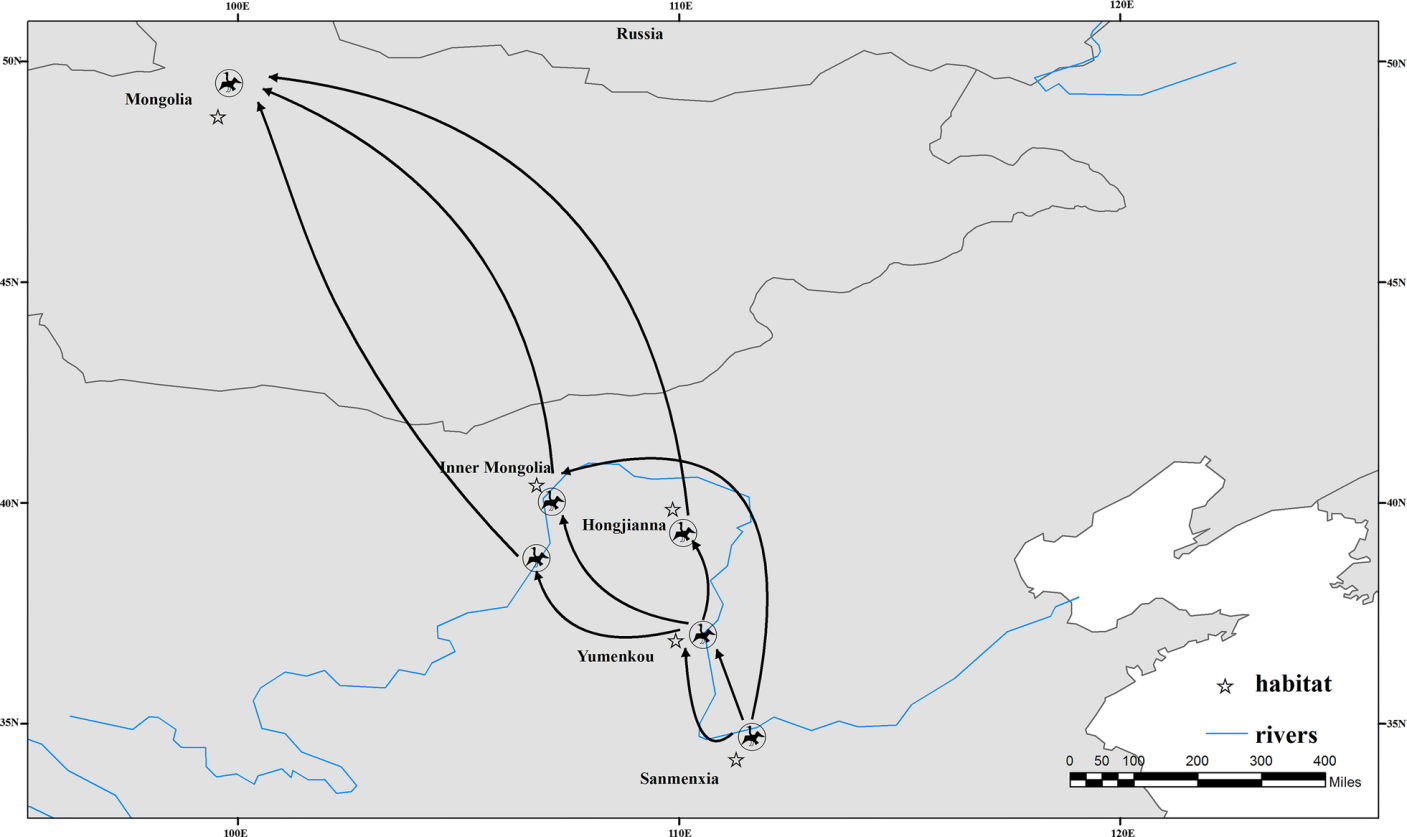
Migration routes of overwintering *Cygnus cygnus* in Sanmenxia, China

The detection of *E. bieneusi* spores in multiple water sources (including irrigation water used for crops, domestic water, and effluents from wastewater treatment plants) supports the idea that water may represent a transmission vehicle for this parasite [20–22]. Based on the report from Li [23], we produced a migration map for *C. cygnus* after they overwintered (Fig. 4), which further illustrates how *E. bieneusi* might spread. Migration is mainly divided into three stages: the first stage is from Sanmenxia to the Yumenkou wetlands, the second is to the Yellow River in Inner Mongolia, and the third is into central and western Mongolia. Zhang et al. [24] followed overwintering *C. cygnus* in Sanmenxia using bird bands and GPS technology, and found that at least 40 bodies of water were associated with overwintering *C. cygnus* in Sanmenxia. Thus, *E. bieneusi* infection in *C. cygnus* may pose a serious threat to humans and other animals in the region. Although better understanding of *E. bieneusi* infection in *C. cygnus* would help with the development of adequate prevention and control strategies against this parasitic infection, further research is needed on tracking the sources of human protozoan pathogens in water bodies. Clearly, regularly surveying harmful microorganisms in water should be a public health priority.

## Conclusions

To the best of our knowledge, the present study is the first to report *E. bieneusi* in *C. cygnus*, and to assess the prevalence of this pathogen in overwintering *C. cygnus* in China. The 11 genotypes identified indicate that the host range for *E. bieneusi* is much wider than previously thought. Importantly, six of the *E. bieneusi* genotypes from this study have been reported in humans, indicating that *C. cygnus* should be considered a potential source of *E. bieneusi* infections in humans and other animals. Therefore, effective strategies should be designed to control *E. bieneusi* infection in *C. cygnus*, other animals, and humans.

## Data Availability

Data supporting the conclusions of this article are included within the article. Nucleotide sequences were deposited in the GenBank database under the accession numbers MN179305-MN179315.

## Abbreviations

ITS: internal transcribed spacer
SSU rRNA: small-subunit ribosomal RNA
SNP: single nucleotide polymorphism
